# Direct venous inoculation of *Plasmodium falciparum* sporozoites for controlled human malaria infection: a dose-finding trial in two centres

**DOI:** 10.1186/s12936-015-0628-0

**Published:** 2015-03-18

**Authors:** Benjamin Mordmüller, Christian Supan, Kim Lee Sim, Gloria P Gómez-Pérez, Carmen Lucelly Ospina Salazar, Jana Held, Stefanie Bolte, Meral Esen, Serena Tschan, Fanny Joanny, Carlos Lamsfus Calle, Sascha JZ Löhr, Albert Lalremruata, Anusha Gunasekera, Eric R James, Peter F Billingsley, Adam Richman, Sumana Chakravarty, Almudena Legarda, Jose Muñoz, Rosa M Antonijoan, Maria Rosa Ballester, Stephen L Hoffman, Pedro L Alonso, Peter G Kremsner

**Affiliations:** Institut für Tropenmedizin, Eberhard Karls Universität Tübingen and German Center for Infection Research, 72074 Tübingen, Germany; Sanaria Inc, Rockville, MD 20850 USA; Barcelona Center for International Health Research (CRESIB), Hospital Clínic, University of Barcelona, E-08036 Barcelona, Spain; Drug Research Center (CIM), Institute of Biomedical Research (IIB), Research Institute of Santa Creu and Sant Pau Hospital, Barcelona, Spain; Department of Pharmacology and Therapeutics, Autonomous University of Barcelona (UAB), Santa Creu and Sant Pau Hospital, E-08026 Barcelona, Spain

**Keywords:** Malaria, *Plasmodium falciparum* sporozoite, Microbial challenge, Controlled human malaria infection, Clinical trial

## Abstract

**Background:**

Controlled human malaria infection (CHMI) accelerates development of anti-malarial interventions. So far, CHMI is done by exposure of volunteers to bites of five mosquitoes carrying *Plasmodium falciparum* sporozoites (PfSPZ), a technique available in only a few centres worldwide. Mosquito-mediated CHMI is logistically complex, exact PfSPZ dosage is impossible and live mosquito-based interventions are not suitable for further clinical development.

**Methods:**

An open-labelled, randomized, dose-finding study in 18–45 year old, healthy, malaria-naïve volunteers was performed to assess if intravenous (IV) injection of 50 to 3,200 aseptic, purified, cryopreserved PfSPZ is safe and achieves infection kinetics comparable to published data of mosquito-mediated CHMI. An independent study site verified the fully infectious dose using direct venous inoculation of PfSPZ. Parasite kinetics were assessed by thick blood smear microscopy and quantitative real time PCR.

**Results:**

IV inoculation with 50, 200, 800, or 3,200 PfSPZ led to parasitaemia in 1/3, 1/3, 7/9, and 9/9 volunteers, respectively. The geometric mean pre-patent period (GMPPP) was 11.2 days (range 10.5–12.5) in the 3,200 PfSPZ IV group. Subsequently, six volunteers received 3,200 PfSPZ by direct venous inoculation at an independent investigational site. All six developed parasitaemia (GMPPP: 11.4 days, range: 10.4–12.3). Inoculation of PfSPZ was safe. Infection rate and pre-patent period depended on dose, and injection of 3,200 PfSPZ led to a GMPPP similar to CHMI with five PfSPZ-infected mosquitoes. The infectious dose of PfSPZ predicted dosage of radiation-attenuated PfSPZ required for successful vaccination.

**Conclusions:**

IV inoculation of PfSPZ is safe, well tolerated and highly reproducible. It shall further accelerate development of anti-malarial interventions through standardization and facilitation of CHMI. Beyond this, rational dose selection for whole PfSPZ-based immunization and complex study designs are now possible.

**Trial registration:**

ClinicalTrials.gov NCT01624961 and NCT01771848.

## Background

Malaria affects almost half of the world’s population and it is estimated that in 2013 584,000 deaths occurred [1]. The size of the problem and the ability of *Plasmodium spp.* to adapt rapidly to man-made interventions require expedited development of new anti-malarial tools [2]. To accelerate clinical development of antimicrobials and vaccines, human challenge models are of particular interest. Controlled human malaria infection (CHMI) with *Plasmodium falciparum* is among the best studied challenge models and has paved the way for many current malaria vaccine candidates [3] and some drugs for treatment and chemoprophylaxis [4]. Perhaps the main advantage of CHMI over studies under natural exposure is that it provides consistent and predictable infections, which translates to the ability to conduct simple, well-controlled trials in a small number of healthy subjects, who do not belong to a vulnerable group. This results in early, well-founded decisions on further clinical development.

CHMI by infected mosquitoes requires the bites of five *P. falciparum* sporozoite (PfSPZ)-infected mosquitoes to achieve consistent transmission, whereas one to two infected mosquitoes produce an infection rate between 50% [5,6] and 83% [7]. This led to the consensus to use five infected mosquitoes for CHMI, a number that rarely fails to induce parasitaemia in malaria-naïve volunteers [5,8] and typically leads to microscopically detectable parasitaemia nine to twelve days after infection (pre-patent period). Depending on the laboratory that produces the PfSPZ-infected mosquitoes numbers required to achieve consistent infection can be lower [7,9]. Length of pre-patency varies considerably between centres [10], which is partly explained by the use of different procedures and parasite isolates. Besides the complexities of maintaining a suitable insectary, major constraints of mosquito-mediated CHMI are the restricted time window during which the mosquitoes can be used for infection, the logistic challenge of having infected mosquitoes and volunteers available at the same time, a large (and largely unknown) biological variability in the number of inoculated parasites, and the need for dissection of mosquitoes after the blood meal to prove infection and blood intake, which may require re-exposure in case the mosquitoes are negative. Direct measurement of the number of mosquito-inoculated PfSPZ in humans is not possible, and variability in pre-patent period, number of mosquitoes required for consistent infection [5,7-9] as well as vaccination success after transmission of attenuated PfSPZ [11,12] suggests that PfSPZ dose is poorly controlled by counting the number of bites or mosquitoes. A potential way to overcome these constraints is injection of purified, cryopreserved, quantitated PfSPZ. In addition, such injectable PfSPZ are being developed and tested as whole-cell vaccines [13,14].

Recently, manufacture of aseptic, vialed, purified, cryopreserved, infectious PfSPZ (PfSPZ Challenge) that meet regulatory standards has been achieved. In four other clinical trials, PfSPZ Challenge was administered as an intradermal (ID) or intramuscular (IM) injection and PfSPZ successfully infected human volunteers at doses between 2,500 and 25,000 PfSPZ [15-18]. However, the pre-patent period was approximately two days longer than after mosquito-mediated CHMI, and with ID administration there was no dose response.

Here, the results of a CHMI study of PfSPZ Challenge administered by intravenous (IV) injection through an indwelling catheter and by direct venous inoculation (DVI) are reported. The objective of the study was to establish the minimal number of PfSPZ required to consistently infect volunteers with a pre-patent period comparable to published data on exposure to the bites of five PfSPZ-infected mosquitoes.

## Methods

### Study design and participants

The study was an open-labelled PfSPZ Challenge IV dose-escalation trial with an ID injection control arm (Tübingen) and a verification group at a second study site (Barcelona) to assess reproducibility of the best IV dose. Volunteers were to be healthy and malaria-naïve individuals between 18 and 45 years of age, who had no history of malaria or long-term residency in a malaria-endemic area, never received an investigational malaria vaccine, were not immunosuppressed, had no acute or chronic infection or other disease, did not abuse alcohol or any other drug and belonged to a low-risk group for cardiac disease [19]. Before enrolment, written informed consent was obtained and understanding of the study and procedures was assessed with a quiz. The study received approval by the ethics committee of the University Clinic and the Medical Faculty of the University of Tübingen, and in Barcelona by the ethics committees of the Hospital Clinic and the Hospital de la Santa Creu i Sant Pau. The study followed the principles of the Declaration of Helsinki in its 6^th^ revision as well as International Conference on Harmonization – Good Clinical Practice (ICH-GCP) guidelines. The study is registered with ClinicalTrials.gov, numbers NCT01624961 and NCT01771848.

In Tübingen, volunteers were randomly assigned to the IV or ID arm by a computer-generated sequence provided in sealed envelopes by a third party on the day of PfSPZ Challenge injection (Day 0). The injections took place between June 12 and September 12, 2012. Safety follow-up of volunteers was done for six months. Dose-escalation of PfSPZ Challenge IV was in four-fold increases, starting with 50 and ending with a maximal dose of 3,200 PfSPZ. At each dose, three volunteers were injected and if 3/3 became parasitaemic within three weeks another six were inoculated with the same dose. The dose was increased to the next level when less than 3/3 or less than 9/9 became parasitaemic. A further dose increase was planned if 9/9 volunteers became parasitaemic but the geometric mean pre-patent period was greater than 12 days. ID injections were given as two separate doses of 50 μL containing 1,250 PfSPZ in the deltoid region of each arm. In Tübingen, PfSPZ Challenge IV was administered as a slow injection of 0.5 mL via an IV catheter preceded and followed by a flush with at least 2 mL of physiological saline. In Barcelona, an IV catheter was inserted in the left arm to serve as emergency access. PfSPZ were administered by direct venous inoculation (DVI) of 0.5 mL parasite suspension into the right arm by venipuncture using a 1 mL syringe with 25 G × 16 mm needle on April 19, 2013.

### PfSPZ Challenge

PfSPZ Challenge contains aseptic, purified, cryopreserved NF54 PfSPZ, isolated from *Anopheles stephensi* mosquitoes, reared and infected under aseptic conditions [14,20]. NF54 is susceptible to all clinically used anti-malarials and has been used extensively in CHMI experiments [8]. PfSPZ Challenge was kept at -195 to -150°C in liquid nitrogen vapour phase. Two separate lots of PfSPZ Challenge, produced 16 months apart (March 2011 and July 2012), were used in the study. PfSPZ Challenge released for clinical use meets quality control specifications including sterility, purity and potency [14,20]. The quality control release and stability programme assessed potency and viability using *in vitro* infection of cultured human hepatocytes (HC-04) and a membrane integrity assay (Table 1), respectively as described [14,20]. Briefly, 50,000 PfSPZ were added to 40,000 HC-04 (1F9) cells and cultured for six days. Late liver stage parasites were detected by staining with a monoclonal antibody against *P. falciparum* merozoite protein 1. Membrane integrity was tested by fluorescence microscopy of PfSPZ following incubation with SYBR green and propidium iodide. Volunteers were inoculated within 30 minutes after thawing of PfSPZ Challenge.

**Table 1:** ***In vitro*** **infectivity to a hepatocyte line (HC-04) (potency) and sporozoite membrane integrity (viability) of the two lots of PfSPZ Challenge**

**A) Tübingen**		
**Release date**	**Potency ± standard deviation (No. of parasites expressing PfMSP-1/well)**	**Viability ± standard deviation**
Fresh*	32.7 ± 1.5 parasites	98.2%
Release**	29.3 ± 3.1 parasites	87.4% ± 5.9%
3 months	27.3 ± 0.6 parasites	84.6% ± 1.9%
6 months	26.7 ± 1.5 parasites	83.6% ± 5.5%
9 months	26.3 ± 2.5 parasites	86.3% ± 6.5%
12 months	27.3 ± 0.6 parasites	86.2% ± 1.3%
Post last clinical dose Tübingen	24.0 ± 1.7 parasites	81.7% ± 2.6%
**B) Barcelona**		
**Release date**	**Potency ± standard deviation (No. of parasites expressing PfMSP-1/well)**	**Viability ± standard deviation**
Fresh*	28.3 ± 1.5 parasites	95.5%
Release**	25.3 ± 1.5 parasites	89% ± 2.2%
3 months	21.7 ± 1.5 parasites	85% ± 3.3%
6 months	25.0 ± 5.3 parasites	86% ± 4.8%
Post last clinical dose Barcelona	19.0 ± 1.0 parasites	85% ± 4.4%

*Fresh refers to the aseptic, purified PfSPZ of this lot before they were cryopreserved. Data from all other time points were generated on thawed PfSPZ Challenge.

**Release refers to the data generated within a few weeks of manufacture that were used to demonstrate that PfSPZ Challenge met quality control “release” specifications. All other data are from the formal stability programme.

### Procedures

All volunteers were observed for at least one hour after PfSPZ Challenge administration (Day 0) and examined on the subsequent day (Day 1) followed by daily telephone or electronic mail contacts. In Tübingen, twice-daily visits and thick blood smears were performed from Day 5 until the first thick blood smear was positive or Day 21 was reached. In Barcelona, once-daily visits were performed between Days 6 and 9, followed by twice-daily visits between Days 10 and 15. Quantitative thick blood films were prepared as described [21] at least once a day. Two or more microscopists were required to observe a minimum of two unambiguous parasites to declare a slide positive with a limit of detection below four parasites per μL. On the day of first microscopically detectable parasitaemia or Day 21 (if no parasites had been detected by then) volunteers started a curative anti-malarial treatment with artemether-lumefantrine (Tübingen) or chloroquine (Barcelona). Subjects were considered cured when two consecutive thick blood smears were negative and symptoms ceased. Later follow-up visits of volunteers occurred on Days 28, 84 and 168 after inoculation in Tübingen, and on Days 35 and 90 in Barcelona. Adverse events (AE) and clinical symptoms were reviewed daily until Day 21 and on all follow-up visits thereafter.

DNA from blood was isolated before PfSPZ Challenge administration and every second day beginning on Day 5 in Tübingen or every time a blood smear was taken to perform a thick blood smear in Barcelona. Parasitaemia was estimated by quantitative polymerase chain reaction (qPCR) as described previously [22]. DNA extraction of blood samples and a dilution from ring stage parasite culture was done in the presence of an extraction control (DEC 610, Bioline) using silica spin columns (Qiagen). Amplification and detection of fluorescence was done with a RotorGene 3000 (Corbett). Limit of quantification of qPCR was 30 parasites per mL. In contrast to thick blood smear, qPCR procedures were not fully validated and were considered exploratory. All qPCR runs were performed after completion of the trial.

### Objectives

The primary objective of the study was to identify a PfSPZ Challenge dose that safely infects 9/9 volunteers after intravenous injection and the secondary objective was to assess if increasing the PfSPZ Challenge dose results in a pre-patent period of twelve or less days. Successful infection was defined as the appearance of asexual parasites in peripheral blood, detected by thick blood film microscopy. Upon completion of the trial in Tübingen it was clear that the objectives regarding infection rate and pre-patent period had been achieved. It was, therefore, decided to verify reproducibility of the successful dose of 3,200 PfSPZ IV in an independent group (n = 6) at a different study site in Barcelona, and to use DVI instead of injection through an in-dwelling catheter to administer PfSPZ Challenge.

### Statistical analysis

A one parameter exponential model was used to model the effect of dose on the probability of infection [23]. The effect of dose on length of pre-patent period was modelled under the assumptions that volunteers who did not develop parasitaemia until Day 21 had no risk of developing parasitaemia thereafter and that the relationship between dose and pre-patent period was linear. Parasitaemia on the day of first positive thick blood smear was used as a covariate in the model. Dose and parasitaemia were log_10_-transformed. PCR data were used to estimate parasite multiplication rates using a mixed linear model with volunteer as random variable and PfSPZ Challenge dose and time as independent variables. Safety and tolerability data were analysed by descriptive and visual methods following published guidelines and grading schemes for clinical and laboratory abnormalities [24]. Calculations were done with R version 2.15.2 [25] and a two-sided p < 0.05 was considered statistically significant.

## Results

### Dose escalation of intravenous PfSPZ Challenge

**Figure 1:**
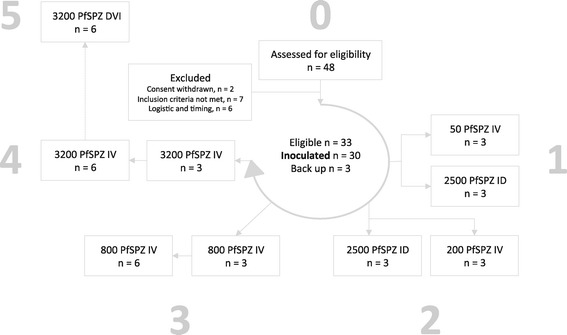
**Trial profile.** In Tübingen the IV dose of PfSPZ Challenge IV was increased sequentially in 4 steps from 50 (1), to 200 (2), to 800 (3) and to 3,200 PfSPZ (4). In steps 1 and 2, volunteers were randomly assigned to receive PfSPZ Challenge ID or IV. An independent verification group (3,200 PfSPZ DVI) in Barcelona was added after completion of the 3,200 PfSPZ IV group in Tübingen.

**Figure 2:**
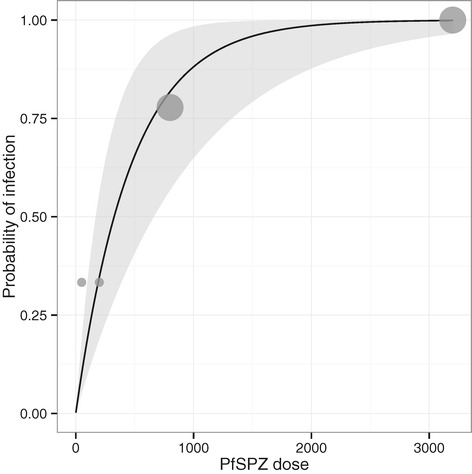
**Effect of IV PfSPZ Challenge dose on probability of infection.** Observed values are given as grey dots, with size representing weight. Model estimates are represented as the black line and the 95% confidence interval as grey ribbon.

A total of 30 volunteers with similar demographic characteristics were included in the dose-escalation phase of the trial (Table 2). Six received 2,500 PfSPZ Challenge ID and 24 IV (Figure 1). PfSPZ Challenge ID led to four successful infections with a geometric mean pre-patent period of 13.6 days (Table 3). The infection rate and pre-patent period of PfSPZ Challenge ID were comparable to what was achieved with 2,500 PfSPZ ID in previous studies [16,15]. The PfSPZ Challenge IV dose was increased sequentially from 50 (n = 3), to 200 (n = 3), to 800 (n = 9) and finally to 3,200 (n = 9) PfSPZ. Injection of 50, 200 and 800 PfSPZ IV led to asexual erythrocytic stage parasitaemia in 1/3, 1/3 and 7/9 volunteers, respectively. In contrast, injection of 3,200 PfSPZ led to asexual erythrocytic stage parasitaemia in 9/9 (100%) volunteers (Table 3). Statistical modelling of probability of infection (Figure 2) with an exponential model [23] estimates an 50% infectious dose of 326 PfSPZ (95% confidence interval [CI]: 169–662).

**Table 2:** **Demographic characteristics of the participants**

**Variable**	**Total**	**ID 2500**	**IV 50**	**IV 200**	**IV 800**	**IV 3200**	**DVI 3200**
N	36	6	3	3	9	9	6
Age in years*	26 (19; 43)	24 (21; 42)	24 (23; 27)	27 (27; 32)	26 (21; 43)	27 (24; 30)	29 (19; 40)
Gender, Female:Male^#^	11:25	1:5	0:3	0:3	4:5	2:7	4:2
Height in cm*	176 (159; 196)	178 (166; 196)	186 (178; 190)	169 (167; 186)	177 (166; 189)	176 (163; 196)	167 (159; 172)
Weight in kg*	73 (55; 111)	83 (63; 92)	77 (70; 100)	77 (68; 82)	74 (59; 90)	71 (64; 111)	61.1 (55; 73)
BMI in kg/m^2^*	23.9 (18.5; 29.4)	24.1 (22.9; 29.4)	22.1 (21.3; 28.9)	24.3 (22.3; 28.7)	23.8 (19.1; 26.6)	24.1 (18.5; 28.8)	22.8 (19.1; 24.8)
Hb in g/dl*	14.7 (11.8; 16.7)	15.1 (12.5; 15.3)	15.6 (15.1; 16.2)	16.1 (14.7; 16.7)	14.2 (12.0; 16.6)	14.6 (11.8; 15.7)	13.3 (12.3; 15.1)
Platelets/nL*	248 (138; 396)	231 (172; 260)	237 (138; 340)	263 (210; 307)	221 (176; 261)	246 (193; 396)	321 (259; 364)

*Median (min; max), ^#^N, ID: intradermal, IV: intravenous (dose-escalation group), DVI: direct venous inoculation (verification group).

**Table 3:** **Infection rate, pre-patent period and time to malaria**

**Group**	**Inoculated (N)**	**Parasitaemic (N)**	Prepatent period in days* (Days)	**Incubation period** ^**‡**^ **(Days)**
ID 2500	6	4	13.6 (12.3 – 15.3)	14.2 (13.0 – 16.0)
IV 50	3	1	13.3 (NA)	7.5 (NA)
IV 200	3	1	13.9 (NA)	15.0 (NA)
IV 800	9	7	11.7 (10.9 – 12.5)	12.0 (11.0 – 13.5)
IV 3200	9	9	11.2 (10.5 – 12.5)	9.5 (7.0 – 12.5)
DVI 3200	6	6	11.4 (10.4 – 12.3)	10.6 (10.0 – 12.0)

*Time from inoculation to first positive thick blood smear, given as geometric mean (min–max).

^‡^Time from inoculation to first symptom judged at least possibly related to malaria, given as geometric mean (min–max).

NA: not applicable, ID: intradermal, IV: intravenous, DVI: direct venous inoculation (verification group).

**Figure 3:**
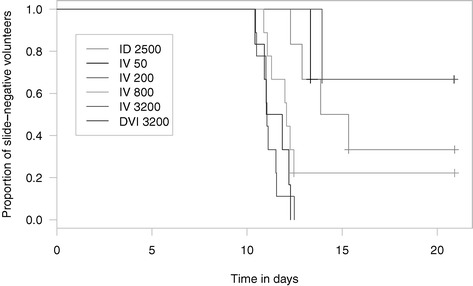
**Kaplan-Meier plot of time to infection.**

**Figure 4:**
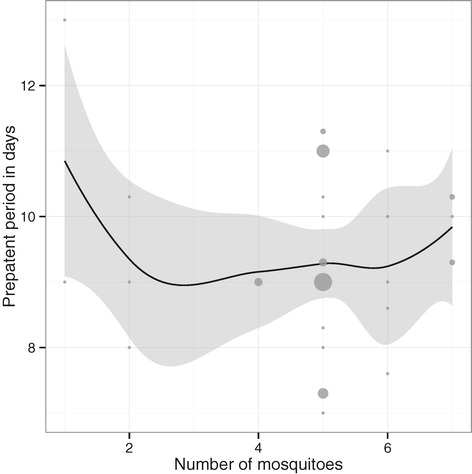
**Effect of number of mosquito bites on pre-patent period.** Published data are from 44 volunteers bitten by 1–7 infected mosquitoes at one centre (Department of Medical Microbiology, Radboud University Nijmegen Medical Centre, Nijmegen, The Netherlands) [26]. Observed values are given as grey dots with size representing weight. A Loess smooth is displayed with the estimate (black line) and 95% confidence interval (grey ribbon).

A geometric mean pre-patent period not longer than twelve days was observed in the 800 PfSPZ (11.7 days) and 3,200 PfSPZ (11.2 days) groups (Figure 3). In the IV groups, dose, corrected for parasite density, explained 73% of variability in pre-patent period and every tenfold increase in dose of PfSPZ led to a 36 (95% CI: 23–48) hours reduction in pre-patent period. This is in contrast to published data [26] on the dose-response relationship between number of mosquitoes and pre-patent period (Figure 4).

### Dose verification

Six volunteers received 3,200 PfSPZ Challenge by DVI by an independent team in Barcelona. All (6/6) became parasitaemic with a geometric mean of 11.4 days (Table 3). All volunteers at both sites were treated with an anti-malarial, either when thick blood smears became positive or on Day 21 in those who still had a negative thick blood smear.

### Early parasite kinetics measured by qPCR

**Figure 5:**
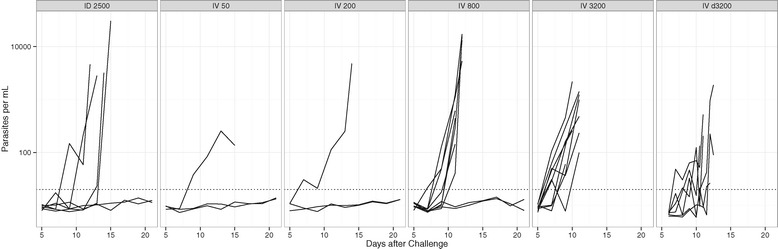
**Parasite quantification by qPCR.** Blood was assessed every other day beginning on Day 5 and on the day of microscopically detected parasitaemia if no sampling was scheduled for that day in Tübingen. In Barcelona blood was sampled every day from Days 6 to 9 and twice daily from Day 9 onward. The dotted line indicates the limit of quantification of qPCR (30 parasites per mL).

All blood smear results were confirmed by qPCR and none of the microscopically negative volunteers reached the positivity threshold. On average, qPCR detected parasites 65 (range 2–167) hours before microscopy. The pattern of parasite multiplication did not show pronounced synchronicity (Figure 5). The estimated parasite multiplication rate per 48 hours until the first positive thick blood smear, was 10.2 (95% CI 5.0–21.0).

### Safety and tolerability of PfSPZ Challenge

**Figure 6:**
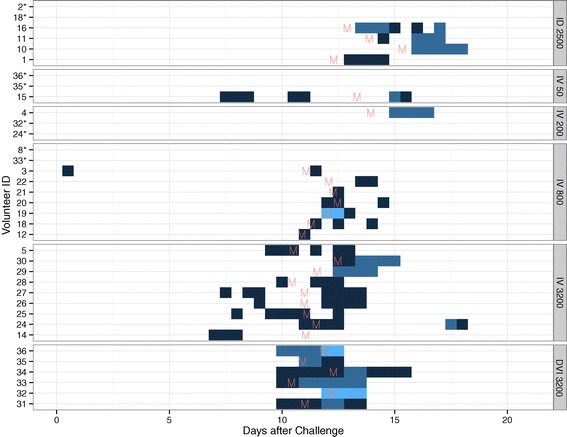
**Adverse event pattern.** Adverse event (AE) episodes are given over time from Day 0 through Day 21. Shades of blue represent AE grading from Grade 1 (dark), to Grade 2 (blue), to Grade 3 (light blue). Volunteer IDs with a star indicate those volunteers who did not develop parasitaemia. The time of parasite detection by microscopy is given as the letter ‘M’ in red.

Overall, IV injection of PfSPZ Challenge through a catheter and by DVI was very well tolerated. Nevertheless, one volunteer (800 PfSPZ group) experienced mild nausea ten hours after injection, which was considered possibly related to PfSPZ Challenge administration. No other individual experienced a PfSPZ injection-related AE. A total of 28 volunteers developed parasitaemia detected by thick blood smear and all developed at least one symptom characteristic of malaria. A total of 286 adverse events (AE) occurred in 34 of the 36 volunteers. The vast majority of AEs happened around the time when parasitaemia became detectable by microscopy (Figure 6). Most AEs were mild (Grade 1; n = 232). Fourteen volunteers experienced moderate (Grade 2) and six severe (Grade 3) AEs, respectively (Table 4). No serious AE (SAE) occurred.

**Table 4:** **Grade 2 and Grade 3 adverse events**

**Group**	Grade 2*	Grade 3*
ID 2500 (n = 6)	Fatigue (2)	None
	Fever (1)	
	Headache (2)	
	Tachycardia (2)	
	High ALT (1)	
IV 50 (n = 3)	Fever (1)	None
IV 200 (n = 3)	Fever (1)	None
	Headache (1)	
	Tachycardia (1)	
IV 800 (n = 9)	None	Lymphopenia (1)
IV 3200 (n = 9)	Fatigue (2)	None
	Fever (2)	
DVI 3200 (n = 6)	Anxiety (2)	High CRP (1)
	Contusion (1)	
	Common cold (1)	
	Fatigue (1)	
	Fever (4)	
		Fever (2)
	Headache (3)	
	High ALT (2)	
	High AST (1)	
	High CRP (1)	
	Insomnia (1)	
	Low potassium (1)	Lymphopenia (3)
	Malaise (1)	
	Menstrual cramps (1)	
	Myalgia (4)	
	Nausea (2)	
	Neutropenia (1)	
	Stye (1)	
	Vomiting (2)	
	Weakness (1)	
Total N	47	7

Note that volunteers in the verification group underwent a more extensive blood-sampling scheme for laboratory parameters. Laboratory parameters were graded using an adapted U.S. FDA toxicity scale.

*Symptom (N), ID: intradermal, IV: intravenous, DVI: direct venous inoculation (verification group).

The most frequent AE was headache (n = 58, 6 Grade 2). This was followed by fatigue (n = 35, 5 Grade 2) and fever or fever-associated symptoms (n = 32, 9 Grade 2, 2 Grade 3). Laboratory abnormalities included liver enzyme increases (n = 7), lymphopenia (n = 8) and bilirubinuria (n = 2). All resolved uneventfully and there were no delayed onset AEs.

## Discussion

Two previous studies have shown that PfSPZ Challenge can infect malaria-naïve volunteers following ID [16] and IM [15] injection but parasite kinetics, number of PfSPZ required and infection rate are different from mosquito-mediated CHMI. The present study found that 3,200 aseptic, purified, cryopreserved PfSPZ administered by IV injection consistently infected all subjects (15/15) with *P. falciparum* malaria and resulted in a pre-patent period of 10.4–12.5 days, which is comparable to the pre-patent period often observed in volunteers exposed to the bites of five PfSPZ (NF54)-infected mosquitoes [13,14,27]. It also demonstrated that the infectivity increased from 33% to 100% and the geometric mean pre-patent period was reduced from >13 days to 11.2 days as the dose of PfSPZ was increased from 50 to 3,200. Importantly, IV administration of PfSPZ Challenge was safe and well tolerated and the results were reproduced at another site that used a different lot of PfSPZ, a different clinical team, and a simplified method of injection (DVI).

Kinetics of mosquito- and IV PfSPZ Challenge-mediated CHMI are very comparable. Mosquitoes deposit PfSPZ in blood vessels and into the skin, where some of them get access to the vascular system [28]. Since it has been suggested that the “skin stage” of malaria has an important role in immunity [29], it would be interesting to compare immune responses to IV and mosquito-mediated *P. falciparum* malaria in direct comparison. However, it should be noted that beginning with the seminal report on protection of mice against malaria by immunization with radiation attenuated sporozoites in 1967 [30], almost all work in animal models to develop and understand radiation attenuated sporozoite-induced immunity has used IV immunization and IV challenge. Furthermore, it has recently been shown that mosquitoes directly cannulate small vessels when feeding [31].

Notwithstanding the “non-natural” mode of administration, several important advantages emanate from the successful translation of mosquito-mediated to mosquito-free inoculation of PfSPZ for CHMI: I) improved standardization, II) exact dosing and dose-estimation for immunization studies and III) facilitated global access to CHMI in times where testing capacities shall be expanded, particularly to endemic regions.

Standardization of CHMI is central for comparison of studies between and within clinical sites and populations. In the current study, this topic was addressed by introduction of an independent verification group, located about 1000 km away, assessed by a different clinical and parasitological team that used DVI instead of IV injection *via* catheter. In addition, two different PfSPZ Challenge lots, produced 16 months apart were used. Despite these obvious differences, results were highly comparable, showing that the technique is not centre-dependent and amenable to multicentre studies, which would be hardly possible using mosquito-mediated CHMI [10].

Conversion of number of mosquitoes into PfSPZ dose cannot be exact. Nevertheless, data of this study helps to improve dose-estimation for immunization trials. Immunization by IV injection of irradiated PfSPZ (PfSPZ Vaccine) requires a cumulative dose of 675,000 PfSPZ to protect 6/6 volunteers, whereas 540,000 PfSPZ provide only partial protection (6/9) [13]. When using infected and irradiated mosquitoes 1,000 bites are needed for protection [12,32]. Hence one mosquito transmits more than 540 and up to 675 PfSPZ Vaccine equivalents. This matches exactly the estimate of the present CHMI study: five mosquitoes with 640 PfSPZ Challenge equivalents per mosquito. The task of rational dosing becomes even more complicated when the biologically more relevant variable “successful passage through the liver” is considered: Assuming a 10.2-fold multiplication of asexual parasites and 10,000 successful erythrocyte invasions per infected hepatocytes in all volunteers of this study, 3,200 PfSPZ successfully infected 13 (95% CI: 8–21) hepatocytes. This extrapolation is likely inaccurate but shall stimulate further research and technological progress, since the infected hepatocyte is a crucial immunogen in PfSPZ-based immunization strategies and hence shall be directly monitored to ensure high-level protection. Results of the first trial, where this *a priori* knowledge has been used to dose PfSPZ Challenge for the chemoprophylaxis with sporozoites approach [33,34], are expected in early 2015 [35].

So far, CHMI with mosquitoes is restricted to a small number of centres globally. Development of a standardized protocol for CHMI with PfSPZ Challenge enables every malaria-experienced centre to perform CHMI studies to assess anti-malarial drugs and vaccines, diagnostics, and innate and acquired resistance to malaria. Due to the high reproducibility and temporal independence, previously unfeasible study designs can be realized; from complex early phase (e.g. sequential and adaptive) to large multicentre trials in populations with very low natural exposure (e.g. travellers and populations in pre-eradication settings) and studies in endemic countries. In fact, the first CHMI trial using PfSPZ Challenge IV is underway in Gabon and several parallel trials are in progress to assess PfSPZ Vaccine IV in the United States, Europe and Africa.

## Conclusions

Exposure to five infected mosquitoes is a standard technique for controlled human malaria infection (CHMI) in humans. Successful translation of mosquito-administered to injectable *Plasmodium falciparum* sporozoite (PfSPZ) mediated CHMI is an important step in standardization and harmonization of CHMI. It allows complex and larger multicentre trials and increases the number of groups with access to CHMI in times of active development of novel preventive and therapeutic interventions. CHMI using intravenous inoculation of PfSPZ Challenge is safe, well tolerated, highly reproducible and shall boost the understanding of malaria and the development of novel anti-malarial interventions.
